# Well-Adhered Copper Nanocubes on Electrospun Polymeric Fibers

**DOI:** 10.3390/nano10101982

**Published:** 2020-10-07

**Authors:** Temitope Q. Aminu, Molly C. Brockway, Jack L. Skinner, David F. Bahr

**Affiliations:** 1 School of Materials Engineering, Purdue University, West Lafayette, IN 47907, USA; taminu@purdue.edu; 2 Mechanical Engineering, Montana Technological University, Butte, MT 59701, USA; mbrockway@mtech.edu (M.C.B.); jskinner@mtech.edu (J.L.S.)

**Keywords:** nanofibers, microfibers, aligned electrospun fibers, nanostructures, copper, adhesion energy, Gibbs–Wulff–Kaischew shape theory, energy release rate

## Abstract

Electrospun polymer fibers can be used as templates for the stabilization of metallic nanostructures, but metallic species and polymer macromolecules generally exhibit weak interfacial adhesion. We have investigated the adhesion of model copper nanocubes on chemically treated aligned electrospun polyacrylonitrile (PAN) fibers based on the introduction of interfacial shear strains through mechanical deformation. The composite structures were subjected to distinct macroscopic tensile strain levels of 7%, 11%, and 14%. The fibers exhibited peculiar deformation behaviors that underscored their disparate strain transfer mechanisms depending on fiber size; nanofibers exhibited multiple necking phenomena, while microfiber deformation proceeded through localized dilatation that resulted in craze (and microcrack) formation. The copper nanocubes exhibited strong adhesion on both fibrous structures at all strain levels tested. Raman spectroscopy suggests chemisorption as the main adhesion mechanism. The interfacial adhesion energy of Cu on these treated PAN nanofibers was estimated using the Gibbs–Wulff–Kaischew shape theory giving a first order approximation of about 1 J/m^2^. A lower bound for the system’s adhesion strength, based on limited measurements of interfacial separation between PAN and Cu using mechanically applied strain, is 0.48 J/m^2^.

## 1. Introduction

The filamentary polymer architectures traditionally engendered by the electrospinning process have enabled the development of functional material systems and devices that are underpinned by design manipulations at micron and nanometric scales. Leveraging the relative ease of polymer chemistry modification, a high surface-area-to-volume ratio as well as process flexibility [1], electrospun fibers have served as matrices for encapsulation of functional organic and inorganic materials [2] and as robust templates for directed growth confinement of nanostructures [3]. Consequently, hybrid materials based on electrospun fibers have been instrumental in the engineering of targeted drug therapies, filtration membranes, scaffold design for regenerative medicine, electromagnetic interference shielding devices, and membrane fuel cell cathodes [4,5,6,7].

Metallic nanostructures are known to possess striking magnetic, thermal, electronic, and surface properties markedly distinct from those of the bulk [8]. However, the efficient and extended utility of these properties can be attenuated by spontaneous aggregation typically encountered in these nanostructures, being a structural response to reduce their surface energy. Polymers have been widely used to hinder aggregation and bestow stability on metallic nanostructures [9]. Hierarchical structures of conformal metallic nanostructures—either as consolidated films or discrete particles—have been successfully immobilized on electrospun nanofibers and microfibers for a broad range of applications [10,11].

In principle, the contrast between the intrinsic strong cohesive energy of metallic species and the weak van der Waals forces that hold together polymer macromolecules strongly influences interfacial interaction, creating weak adhesion in most metal–polymer systems [12]. As a result, in the context of metallic nanostructures on electrospun fibers, procedures including wet chemical treatments [3] and plasma bombardment [13] that impart functional chemical groups as well as surface micro-roughness have been deployed to improve adhesion at the metal–polymer fiber interface. Despite the importance of a strong interfacial adhesion on structural integrity and material performance, systematic empirical evaluation of adhesion of metallic nanostructures on electrospun fibers in the literature is surprisingly scant. Sonication [3,14], peel tests [15], and inductively coupled mass spectrometry of resultant suspensions after mat immersion [16] have been utilized as measures to assess nanoparticle adhesion or debonding. In the broader research schemes in which these adhesion tests are typically performed, the results are understandably interpreted on a pass or no pass basis based on global or aggregate behavior of the metallic nanostructures on the non-woven fiber architectures. For applications in which the electrospun structures are subjected to mechanical strain (i.e., when being used as water filters where flow causes non-woven membranes to flex) poorly adhered nanoparticles can cause the structure to lose efficacy and lead to potential release of nanoparticles into the environment. Therefore, creating both the structure with well adhered nanoparticles of metal and developing a quantitative assessment of the adhesion of said structures will enable improved design for reliability in these systems.

In this paper, we utilize a bottom-up method to grow well-defined, discretely distributed copper nanocubes on aligned polyacrylonitrile (PAN) nanofibers and microfibers based on electroless deposition from aqueous solutions. The interesting mix of hydrophilicity, chemical stability, and strong mechanical performance of electrospun PAN fibers has enabled their widespread adoption and adaptability as functional scaffolds for diverse application designs [17,18]. Furthermore, metallic nanocubes classically possess a high surface area harnessed for catalysis, and through anchoring on electrospun fibers, a surface area synergy is achieved that enhances functional efficacy [19,20]. More pertinently, the cubic crystal habit represents a model nanostructure for the study of interfacial adhesion between a polymer fiber and a metallic particle due to its shape simplicity and a defined contact area that aids microstructural evaluation at the interface. Nominally, copper has a low reactivity that precludes the formation of strong adhesion on untreated polymeric surfaces [12], so fibers were subjected to chemical treatment based on alkaline hydrolysis. Uniaxial tensile tests are conducted at distinct strain levels to systematically introduce shear stress states at the cube–fiber interface. Raman spectroscopy is conducted to investigate modifications in fiber chemistry due to the metallization process, providing insights into likely mechanisms for adhesion. Lastly, we make an approximation of the adhesion energy of the copper nanocubes on the fibers using geometric relationships based on the Gibbs–Wulff–Kaischew model and energy release rates during crack propagation.

## 2. Materials and Methods

**Figure 1:**
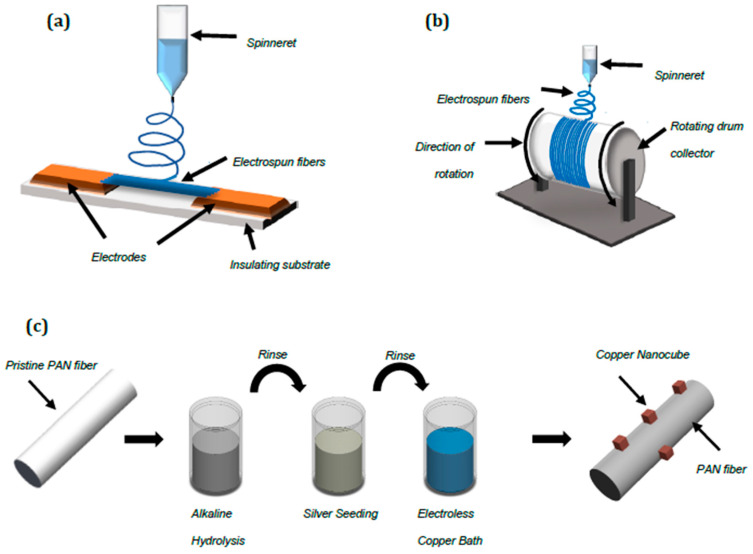
(**a**) Parallel electrode configuration for polyacrylonitrile (PAN) aligned nanofiber processing. (**b**) Rotating drum configuration for PAN aligned microfiber processing. (**c**) Schematic of the electroless deposition procedure for nanocube growth.

Nanometric aligned PAN fibers were electrospun from a 10 wt.% PAN (M_W_ = 150,000, Sigma-Aldrich, St. Louis, MI, USA)/1 wt.% acetone (>99.5%, Sigma-Aldrich, St. Louis, MI, USA) solution in *N*,*N*-dimethylformamide (DMF, >99.8%, Sigma-Aldrich, St. Louis, MI, USA) using a Spraybase vertical electrospinner configuration with parallel electrodes. The solution was dispensed through a 24-gauge blunt needle at a flowrate of 0.20 mL/h towards grounded electrodes in a configuration, as demonstrated in Figure 1a. The needle was held at 7.6 kV at a separation distance of 4.5 cm from the substrate. Deposition proceeded for 10 min at a time before careful transfer to circular nylon washers (ID: 14 mm; ED: 22 mm), which offered a robust support structure for the fiber mats. Aligned polyacrylonitrile (PAN) microfibers were prepared from a 12 wt.% PAN solution/1 wt.% acetone. Fibers were electrospun from a 24-gauge blunt needle held at a separation distance of 11 cm from a rotating drum collector, shown schematically in Figure 1b. Solution flowrate was maintained at 0.30 mL/h for stable electrospinning, with the needle at +2.28 kV and the drum at −2.58 kV. The drum was rotated at 1800 rpm to align the fibers as they were electrospun. Deposition proceeded for 20 min before transfer of fiber mats to nylon washers.

### 2.1. Nanocube Growth on Aligned Fiber Mats

The fibers were permanently affixed to the nylon washers (22 mm × 14 mm × 1.2 mm) with epoxy glue and allowed to cure for 24 h. To mitigate or eliminate handling-induced fiber deformation during the deposition procedure, samples were affixed to L-shaped strips wrapped with carbon adhesive tape that enabled easy transportation between baths. The deposition protocol is shown schematically in Figure 1c. First, samples were cleaned in a 1.63 M solution of sodium carbonate (Na_2_CO_3_) for three minutes. Next, samples were immersed in 1 M solution of sodium hydroxide (NaOH) at a temperature between 45–50 °C for fifteen minutes. The aligned fibers were seeded with silver species to catalyze subsequent copper deposition. An amount of 200 µL of ammonia solution (NH_4_OH) was added to a 10 mL solution of 0.01 M AgNO_3_ under constant stirring. Lastly, a 5 mL solution containing 10 wt.% glucose was added to the solution and stirred for 1 min. To prevent possible photocatalytic reduction of silver, the reaction vessel was wrapped with aluminum foil. Samples were immersed in the silver baths for 1 min and subsequently rinsed with a copious amount of deionized water. There was no apparent change in color or translucence of the samples (due to low number density of fibers). A fresh silver bath was prepared for each sample. All seeding baths were operated at room temperature under quiescent conditions. The chemicals used were reagent grade.

### 2.2. Electroless Copper Deposition

0.1 g of copper salt (CuSO_4_.5H_2_0) and 0.6 g of disodium ethylene diamine tetraacetate (Na_2_H_2_EDTA) were dissolved and mixed in 20 mL of deionized water. An amount of 280 μL of formaldehyde (HCHO) was added into the solution. Drops of sodium hydroxide solution (0.92 g NaOH + 20 mL H_2_O) were pipetted into the solution to adjust the pH of the electroless bath to ∼12.5, measured using a pH meter (AB15, Thermo fisher scientific). The silver-seeded fibers were immersed for 15 min at room temperature also under quiescent conditions. The clear blue color of the bath gradually turned pale green, which subsequently became deep green with attendant turbidity, signifying homogenous precipitation of copper in the solution. Afterwards, samples were rinsed in deionized water and air-dried. A fresh bath was prepared for each sample.

### 2.3. Tensile Testing

Uniaxial tensile tests were carried out on an Admet load frame (Model No. 7603) at a cross head speed of 0.5 mm/min in displacement control. The samples (the washers and fiber mats) were gripped by specialized serrated jaws to prevent slippage during tensile loading. Nominal gage length of the samples was 14 mm. Interfacial adhesion under distinct strain levels of 7%, 11% and 14% strain was systematically investigated. After each test, samples were extracted and prepared for imaging in a methodical manner.

### 2.4. Characterization

Post mortem imaging was carried out on the FEI Nova NanoSEM scanning electron microscope equipped with an Everhart-Thornley detector (ETD) operated in secondary electron detection mode. Prior to imaging, all samples were sputter-coated with a thin (≈2 nm) layer of platinum using a Cressington (208HR) sputter coater at a plasma current of 40 mA for 60 s. Fiber dimensions and orientation distribution were measured using ImageJ (National Institute of Health, MD, USA). Energy dispersive spectroscopy (EDS) was performed on the same microscope using an Oxford INCA energy system with a 30 mm window. Raman spectroscopy was conducted to provide insights into modifications in fiber chemistry and its implications for interfacial adhesion between the nanocubes and PAN fibers; Raman shifts were collected using a Renishaw inVia Raman microscope with a 532 nm laser (at 1% power) with a grating of 2400 L/mm and an objective lens of 100×.

## 3. Results and Discussion

**Figure 2:**
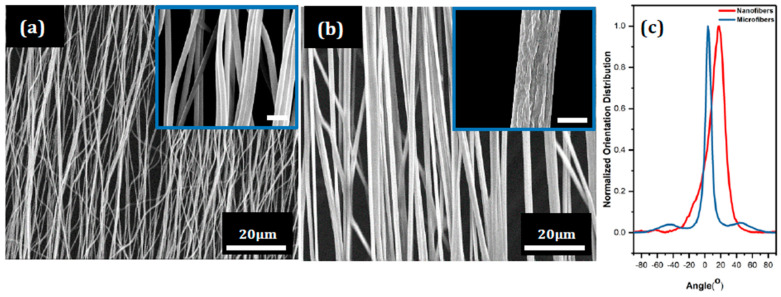
As-spun aligned polyacrylonitrile fibers. (**a**) Nanofibers. Inset: higher magnification. (**b**) Microfibers. Inset: higher magnification. (**c**) Orientation distribution of both nanofibers and microfibers. Size of scale bars in inset images is 1 μm.

Both electrospinning configurations for fiber alignment, that is, parallel electrode and rotating drum configurations, yielded good directionality as shown in microstructures, Figure 2a,b, for the nanofibers and microfibers, respectively. The orientation distribution for both sets of fibers are shown in Figure 2c. Topographically, the nanofibers had a smooth surface (inset Figure 2a); on the other hand, the microfibers exhibited ridge-like surface relief, likely artefacts from the solvent evaporation process (inset Figure 2b).

Both fiber cross-sections were uniform along fiber length, with no occurrence of beads. Average fiber diameters and standard deviation for the nanofibers and microfibers were 336 ± 46 nm and 1.086 ± 0.103 μm, respectively. The distribution’s sharp peak about the median angles (nominally slightly displaced from zero) for both microfibers and nanofibers is indicative of good alignment. Taking into consideration these angular offsets, approximately 66% of the nanofibers and 97% of the microfibers were within ± 10° of their major axial orientation. These estimates were made based on corresponding area fractions in the distribution curve. The greater deviation in alignment observed in the nanofibers can be attributed to a low flexural resistance, which is a function of their thin cross-sections, inducing much greater angular offsets than seen in the microfibers; indeed, global alignment is apparent, but local curvatures in individual fibers impede full rectilinear lengths as displayed in the microfibers.

**Figure 3:**
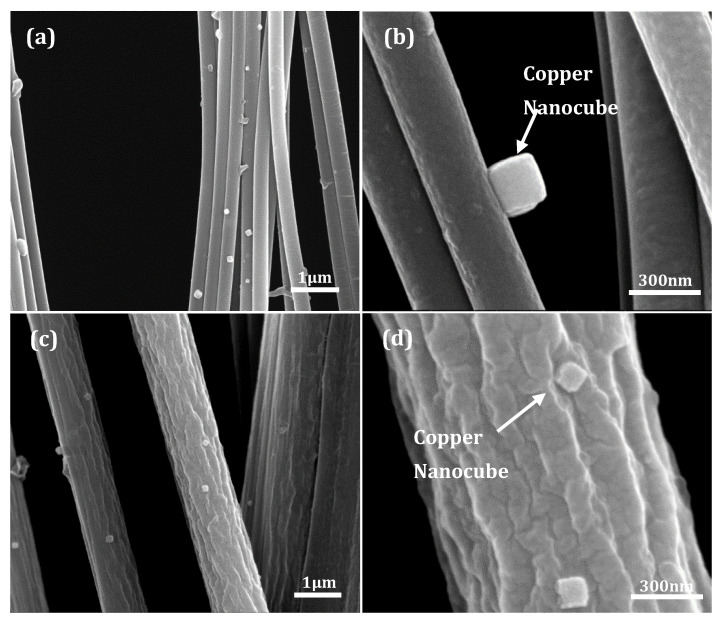
(**a**) Dispersed positions of nanocubes on nanofibers. (**b**) Higher magnification micrograph of a well-defined nanocubes on nanofiber showing cube face anchored to the fiber surface. (**c**) Dispersed distribution of nanocubes on microfibers. (**d**) Higher magnification micrographs of nanocubes anchored to the surface of a single microfiber.

Metastable electroless solutions offer flexibility in compositional control, operational parameters, morphology, and size modulation of nanocrystals in metal deposition procedures. In addition, the necessary immersion of substrates into the solution aids homogenous deposition—of discrete nanoparticles or consolidated nanoparticle films—for a non-planar substrate geometry as presented by electrospun polymer fibers. Figure 3a,c show the evolution of well-defined copper nanocubes on the nanofibers and microfibers, respectively. In addition to their slightly truncated edges or rounded corners, the nanocubes had a disperse distribution on the PAN fibers. Average edge lengths of the nanocubes on the nanofibers and microfibers were 137 ± 39 nm and 124 ± 27 nm, respectively (size distribution shown in Figure S1, Supplementary Information). However, this difference is not statistically significant given a *p*-value > 0.05—see Table S1 for a summary of the statistical analysis. The cube edges not orthogonal to the fiber surface are used for the average edge length computation. The significance of this will be discussed in later sections. High magnification elevation view (of nanocubes on nanofibers) and planar view (of nanocubes on microfibers) are shown in Figure 3b,d, respectively.

Although the nanocubes predominantly evolved with a cube face contacting the supporting PAN fibers, the intrinsic cylindrical shape of the fibers altered the original planar geometry of a cubic facet, introducing commensurate curvature (shown more clearly in Figure S2, supplementary information). However, this effect is considerably less pronounced on the microfibers due to their greater cross-section in relation to the edge lengths of the cubes. Based on particle count, the nanocube morphology accounted for 60–65% of all grown nanostructures on the PAN fibers; the remaining structures were a mix of rose-petal, cauliflower, and bulbous shapes that formed as a result of secondary nucleation on the facets of progenitor cubic crystals

In terms of the thermodynamics of crystal shape and growth, the surface energy of low-index crystal facets typically dictates the resulting crystal habit or morphology [21]. Consequently, based on the energetic sequence $\gamma^{\left\{111\right\}}<\;\gamma^{\left\{100\right\}}<\;\gamma^{\left\{110\right\}}$, the equilibrium shape of single crystals should either have a full octahedral shape to maximize the manifestation of {111} facets [21] or have a truncated octahedral shape based on the coevolution of {111} and {100} facets [22]. However, solution-based deposition processes classically enable control over final crystal morphology through selective stabilization of specific crystal facets with designated capping agents or surfactants [23,24]. With respect to our experiment, the cubic crystal habit, composed of {100} facets, was obtained without the use of exogenous capping agents. While primarily aiding the complexation of copper ions (${\mathrm{Cu}}^{2+}$) in the electroless bath to mitigate spontaneous precipitation of these species in the bulk of the solution, the carboxylate functional groups present in the EDTA molecule have also been proposed to preferentially interact with the {100} facets, inhibiting growth in the <100> directions relative to the <111> directions and effectively constraining the final crystal morphology to a cubic shape [25]. Additionally, by virtue of the conformation of the copper nanocube interface to the substrate curvature, it is conceivable that cubic crystal evolution on the fibers is achieved through a concerted stabilization process that involves active surface species derived from prior chemical treatment.

**Figure 4:**
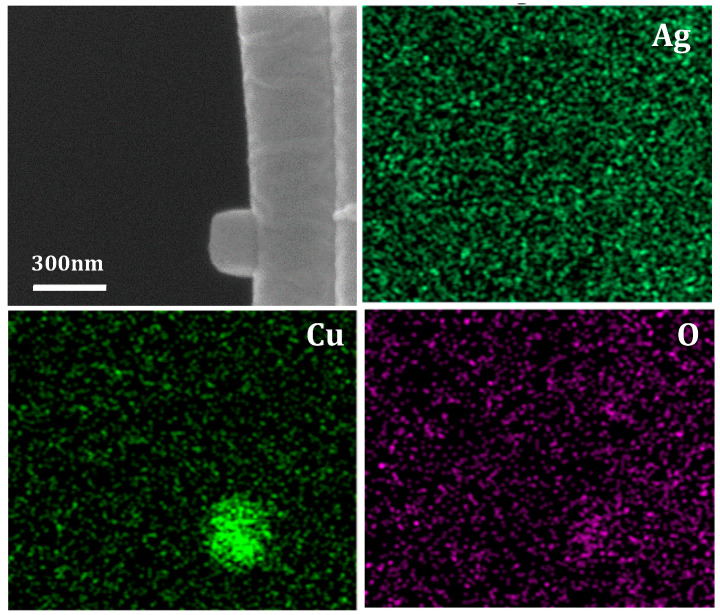
SEM and EDS mapping of copper nanocubes on aligned nanofibers.

Energy dispersive spectroscopy (EDS) was performed for elemental identification. Figure 4 shows elemental maps derived from characteristic X-ray lines for Ag (Lα1), Cu (Kα1), and O (Kα1). The elemental maps show that the cubic structures are indeed composed of copper species. In addition, x-ray diffraction data from preliminary studies (not included) identified the nanoparticulate crystalline phase of pure copper. Furthermore, the short deposition time for the silver seeding process ensured the precipitation of sparse and extremely small catalytic silver seeds that are below the resolution limit of the SEM.

### 3.1. Fiber Deformation and Copper Nanocube Adhesion

**Figure 5:**
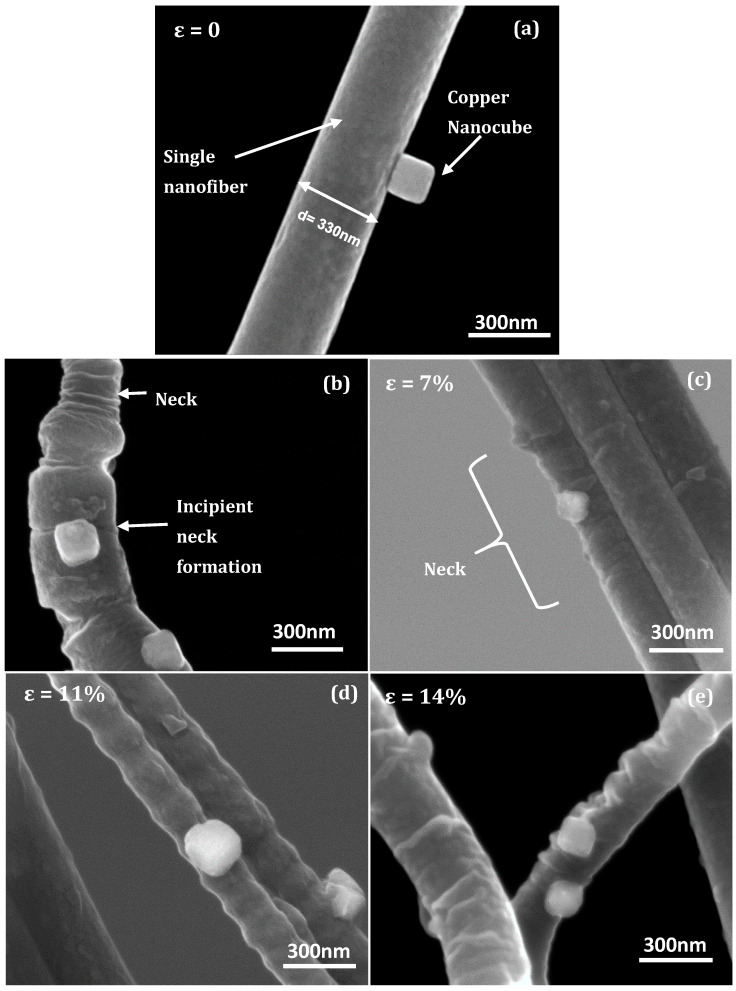
Representative microstructures for (**a**) a copper nanocube on a single nanofiber at zero strain; (**b**) incipient neck region showing nanocubes in vicinity of surface crack; (**c**) nanocube anchored to underlying neck region at ε = 7%; (**d**) adhered nanocubes on neck region at ε = 11%; (**e**) adhered nanocubes on neck region at ε = 14%.

Traditionally, when a metal film is supported by a polymer substrate, and the resulting composite structure is subjected to a tensile load, the more compliant polymer material can suppress strain localization, a prelude to delamination, in the metal film, ultimately facilitating a congruous deformation behavior [26]. Alternatively, the compliant substrate can “shield” a stiff material by absorbing most of the deformation strain if they are attached as islands rather than conformal films [27]. The copper nanocubes grown on the PAN fibers approximate the latter case. Additionally, electrospun fibers present a distinct geometry as well as special size-dependent micromechanical deformation characteristics [28]. Because of the expected discrepancy in induced strain coupled with the aforementioned “shielding” effect, integrity of interfacial adhesion is necessarily evaluated in regions with demonstrably high strain concentrations, i.e., necks and the immediate vicinities of surface microcracks or tears. Figure 5 shows representative micrographs of copper nanocubes anchored to the surface of the nanofibers at the different strain levels. Firstly, the electrospun PAN nanofibers accommodated multiple neck regions at all induced strain levels, indicating an intermittent occurrence of surface instabilities [29]. However, in contrast to macroscale deformation, extensive propagation of each necked region is restricted by adjacent necks [30]. The distance between necks and neck amplitude—calculated as half the difference between average fiber diameter and fiber diameter at the neck—ranged from 90–280 nm and 70–300 nm, respectively. Figure 5a shows a typical copper nanocube anchored to a nanofiber at zero applied strain. Figure 5b provides a general illustration of incipient neck formation in the nanofibers: a circumferential discontinuity or microcrack precedes neck formation and elongation. Moreover, this occurs in close proximity to a nanocube, and it is not improbable that the nanocube is in a high strain field of the fiber substrate. However, this effect might be negated by possible relaxation of surface layer macromolecules aided by their greater chain mobility due to less kinematic hindrance from entanglements [31]. Figure 5c shows deformation at 7% strain where a copper nanocube is firmly anchored to a visibly necked region, with no apparent signs of delamination. Previous work on deformation of a single PAN nanofiber established the onset of plastic deformation to be between 5–10% engineering strain [29]. At a higher strain of 11%—see Figure 5d—substantial reduction of the nanofiber cross-section (d ∼186 nm) can be seen, coupled with periodic undulation as a result of multiple neck formation. Remarkably, the copper nanocubes maintained contact, indicating good adhesion despite possibly enhanced interfacial shear from induced strain mismatch. Nevertheless, a closer inspection of the microstructure reveals the presence of an arrested interfacial crack that apparently propagated from a cube edge. An approximate elevation view of this observation will be introduced in later sections on the computation of interfacial adhesion energy where it is germane to the discussion and analysis. Applied strains of 14%—see Figure 5e—did not necessarily translate to thinner nanofiber cross-sections, which would have aided evaluation at even greater interfacial shear or at extended interfacial crack lengths; instead, more necking regions were formed along the nanofiber length. Nonetheless, the copper nanocubes overall exhibited good adhesion, underscoring a robust interface. We must note here that due to the inevitable misalignments in the nanofibers with respect to the loading axis, the magnitude of localized strain in the necked regions may differ from the stated global or far-field strains. Additionally, the extensive (mm) gage length meant that regions of strain concentration and attendant multiple necking phenomena were interspersed with undeformed fiber sections across the nanofiber length.

**Figure 6:**
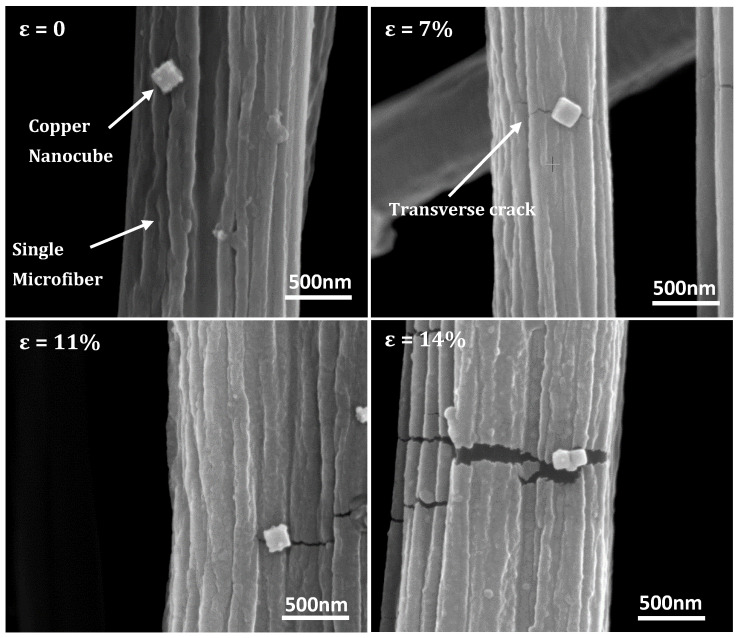
Representative microstructures for: (**a**) PAN microfiber at zero strain; (**b**) ε = 7%; (**c**) adhered nanocubes in path of advancing transverse crack at ε = 11%; (**d**) adhered nanocubes at ε = 14%.

Differences in polymer macromolecule configuration in PAN microfibers and nanofibers influences overall micromechanical deformation behavior or features with respect to stress/strain transmission during tensile loading. Chiefly, because their higher aspect ratio makes them more amenable to applied electrostatic drawing effects during processing, nanofibers possess an enhanced chain orientation or alignment along the fiber axis to a greater extent than microfibers [32,33,34]. Figure 6 shows representative microstructures of copper nanocubes on microfibers at the different strain levels.

With respect to fiber deformation, the microfibers did not exhibit necking phenomena during tensile loading, in contrast to observations of the nanofibers. Rather, craze formation (Figure S3, Supplementary Information) and attendant transverse tears (or microcracks) from strain accumulation were observed to have been sporadically distributed, approximating bulk deformation behavior. However, slight reductions in microfiber diameter may have been counteracted by elastic recovery prior to SEM imaging. It has been proposed that the greater lateral entanglements in bulk polymers inhibits chain ductility, causing local dilatations that ultimately transform into crazes [35]. Consequently, plastic deformation in single microfibers is substantially reduced or restricted [29]. Based on this limitation in plastic flow, we posit that interfacial shear, in comparison to that in the nanofibers, is significantly diminished. Notwithstanding, Figure 6a shows a representative microstructure of a cube-supporting microfiber at zero strain. A typical microstructure at 7% applied strain is shown in Figure 6b, where the propagation of a transverse microcrack through an interfacial area is evident. In theory, the presence or evolution of microcracks at an interface portend a weakening adhesion, which may lead to delamination or debonding events [36]. Empirically, crack formation proceeds from fiber surface, and then propagates through fiber thickness—see Figure S3a,b, Supplementary Information. At strains of 11%—see Figure 6c—pronounced interfacial microcrack is observed, with nanocube contact still preserved. Striking adhesion of contiguous copper nanocubes at the edge of a fully developed microcrack “precipice” derived from an originally intact interfacial area is observed in Figure 6d, for an applied strain of 14%, signifying well adhered nanostructures.

### 3.2. Raman Spectroscopy

**Figure 7:**
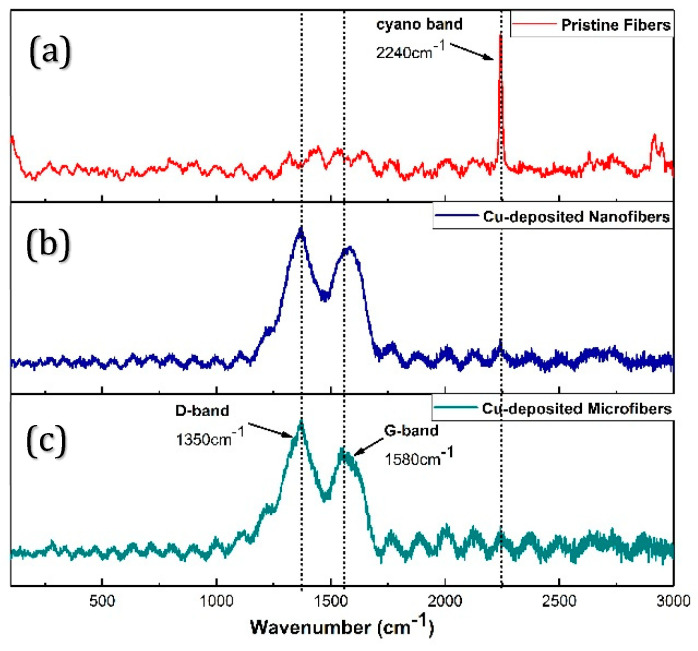
Raman spectra of PAN fibers. (**a**) Pristine fibers. (**b**) Nanofibers after copper nanocube deposition. (**c**) Microfibers after deposition.

Given the strong interfacial adhesion of the nanocubes on both fiber architectures, Raman spectroscopy was used to probe possible modifications in fiber surface chemistry after copper nanocube deposition. The obtained spectra are shown in Figure 7.

The spectrum for pristine fibers is shown in Figure 7a, exhibiting a single sharp, intense peak at a wavenumber of 2240 cm^−1^. This is also an IR active band, indicative of cyano functional groups [37]. Spectra for both nanofibers and microfibers after deposition are shown in Figure 7b,c. Broad and partially conflated bands at 1350 cm^−1^ and 1580 cm^−1^ can be seen, respectively classified as the D and G bands of carbonaceous materials [38]. At the same time, the cyano-band is dramatically reduced in both structures. The G-band is indicative of the presence of graphitic structures from *sp^2^* bonded atoms in ring and chain configuration on the fiber surface, suggesting a conversion of initial linear or aliphatic PAN chain segments to cyclical structures; while the D-band is indicative of the presence of structural disorganization or the existence of foreign atoms (or molecular entities) in surface PAN macromolecules [38,39,40]. Furthermore, based on the peak heights, the band ratio (*I_D_/I_G_*), for the nanofibers and microfibers is 1.15 and 1.30, respectively, indicating that the PAN macromolecules on the microfiber surface possess a slightly higher degree of disorganization than the nanofibers.

Altogether, these chemical and structural changes imply the existence of strong chemical bonds at the nanocube-fiber interface, facilitating the strong adhesion observed in both nanofibers and microfibers. It is also plausible that the bottom-up synthesis approach for the cubic nanostructure formation helped optimize chemical interaction between the treated fiber surface and evolving crystals during growth.

### 3.3. Interfacial Adhesion Energy

#### 3.3.1. Strain Energy Release Model

**Figure 8:**
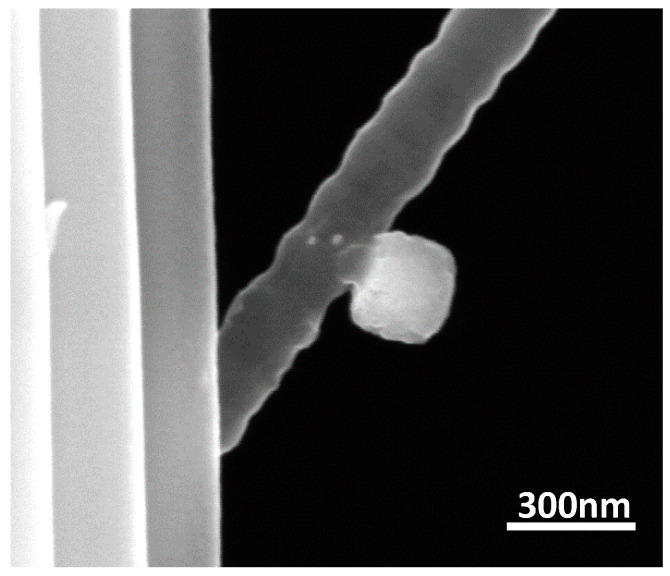
Approximate asymmetric interfacial arrested crack between a copper nanocube and a necked PAN nanofiber at ε = 11%.

The elastic mismatch between a stiff material affixed on flexible substrate imposes shear stress states at the interface that can induce the nucleation and propagation of cracks. Figure 8 shows the elevation view of a partially debonded copper nanocube in the necked region with an arrested interfacial crack at applied nominal strains of 11%.

**Figure 9:**
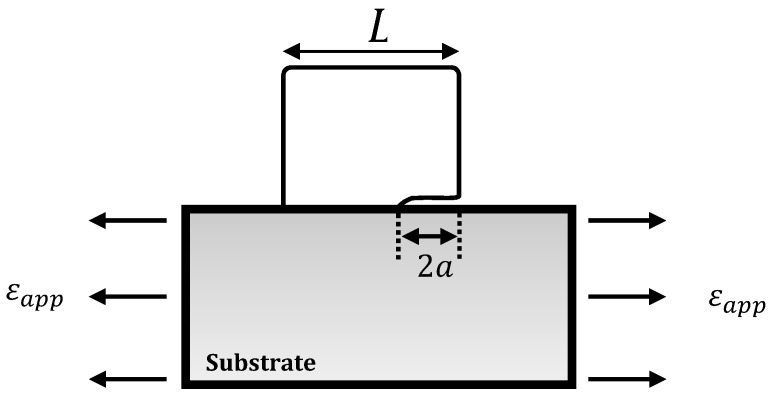
Schematic of asymmetric debonding of a cube “island” on a compliant or soft substrate under the assumptions of plain strain conditions adapted from [41].

Based on the mechanics of interfacial fracture, a resultant crack length upon debonding can be used to approximate the adhesion energy following an analytical model developed by Sun [41] for the estimation of interfacial fracture energy between stiff island structures and a soft substrate. A schematic for an asymmetric debonding event is depicted in Figure 9, wherein a single debond crack propagates from one end of an island edge. Equation 1 expresses the accompanying strain energy release rate, G, as a function of the island width, L, the strain applied to the substrate, $\varepsilon_{app}$, and the interfacial crack length, a. $E_{s}^{*}{\;\mathrm{and}\;E}_{f}^{*}$ are the plane strain Young’s modulus for the substrate and film, respectively

(1) $$G=\frac{\pi }{16}{\left(\varepsilon_{app}\right)}^{2}\left(L-2a\right){\left(\frac{1}{E_{s}^{*}}+\frac{1}{E_{f}^{*}}\right)}^{-1}$$

First, the energy release rate of a crack represents the total energy released during crack propagation per unit increase in crack size [42]. In other words, the release rate represents the dissipation of elastic strain energy in a material or at an interface as a consequence of crack growth. Specifically, the marked distinction in the stiffness of the PAN nanofibers and copper nanocubes gives rise to a non-steady-state condition wherein the energy release rate is dependent on the length of interfacial crack [27]. As a result, at critical applied strains, the energy release rate firstly attains a maximum value at attendant crack length that are substantially smaller than characteristic island size or dimension. The energy release rate decreases with crack growth until it becomes lower than the interfacial fracture energy at which point delamination or debonding ceases [41,43]. Because of this relationship between the crack length and energy release rate, an approximation of the adhesion energy can be made using the model in Equation (1).

An estimate of the adhesion energy was made based on microstructural evidence for nanocube delamination at an applied global strain of 11% as provided by Figure 8, and the following input parameters: nanocube length of 146 ± 10 nm, reported PAN nanofiber modulus of 3 GPa [44], copper modulus of 117 GPa [45], Poisson ratio of 0.3, and a measured crack length, 2a = 83 ± 4 nm. The resulting adhesion energy is 0.48 ± 0.04 J/m^2^, where the standard deviation represents uncertainties resulting from cube and crack dimension measurements. The limitations of identifying individual particles that fit this geometry at any given strain make reproducible measurements of this method challenging.

#### 3.3.2. Gibbs–Wulff–Kaischew Shape Theory for Quantifying Adhesion

**Figure 10:**
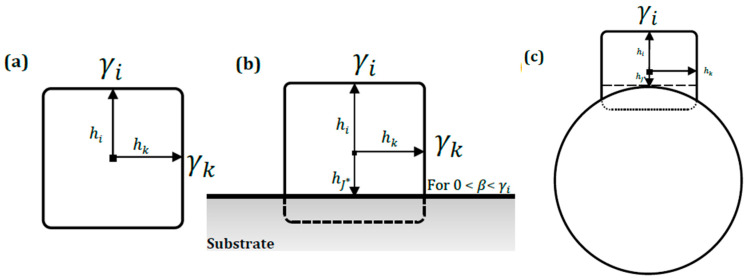
Idealized 2D representation of equilibrium shape of a single cubic copper nanostructure: (**a**) as free crystal according to the Gibbs–Wulff theory; (**b**) on a planar substrate as proposed in the Gibbs–Wulff–Kaischew shape theory; (**c**) on a curved fiber surface.

Alternatively, an approximation of the adhesion energy can be obtained through analysis of resultant particle shapes taking into consideration that these shapes are derived from the equilibration of surface free energies with the immediate microenvironment, including the substrate. This method has the advantage of having many particles, which can be evaluated under identical deposition conditions. Firstly, at a constant volume, the final particle shape should be derived from minimization of the total surface free energy [46]. Accordingly, the Gibbs–Wulff theory states that for a free or isolated crystal at equilibrium, the distance of the center of each bounding facet to an arbitrary central point (Wulff point) within the crystal volume is proportional to the corresponding specific surface energy of that facet. In other words, $\;\gamma_{i}/h_{i}$ = constant (where $\gamma_{i}$ = specific surface free energy of a crystal face *i*, and $h_{i}$ = distance of face center to the Wulff point, Figure 10a) [47]. However, crystal surface energetics, and by extension, final equilibrium shape is modified when the crystal is in contact (i.e., deposited or grown) with a foreign substrate [46]. Consequently, incorporation of the influence of the substrate into the Gibbs–Wulff shape theory was addressed in the unified Gibbs–Wulff–Kaischew theory [46]. In brief, crystal shape on a substrate is effectively truncated through its thickness by a measure proportional to the specific adhesion or interfacial energy (*β*), depicted in Figure 10b. Therefore, the adjusted proportionality given by the substrate–particle interaction is expressed in Equation (2):

(2) $$\frac{\gamma_{i}}{h_{i}}=\;\frac{\gamma_{j}\;-\;\;\beta }{h_{j^{*}}}=constant$$

where $h_{j^{*}}$ = distance of the Wulff point to a planar substrate–crystal interface j* taken to be parallel to a crystal plane *j,* which in turn holds a parallel relationship with the top equilibrium face *i*. $\gamma_{j}$ is the specific surface energy of a face parallel to contact face j*. Consequently, as the adhesion energy increases, crystal truncation increases and vice versa [48]. A useful analogy is the systematic truncation of spherical liquid droplets on solid substrates as the wetting behavior or adhesion increases, reflected by the contact angle in the classical Young’s equation. This simple model provides a geometric framework for a quantitative approximation of the adhesion energy of a cubic crystal structure on a substrate, where, due to its geometric simplicity, the cube distances $h_{i}$ and $h_{j^{*}}$ are readily expressed as functions of measurable cube dimensions. However, in the context of the copper nanocubes on PAN fibers, the fiber curvature influences shape of the interface, creating a non-planar geometry, as shown schematically in Figure 10c. An apparent implication of this curvature is that the contact interface is not strictly parallel to a *j*-th plane in the cubic nanostructures. Hence, in our analysis, given the relatively shallow curvatures, we have assumed a proximate crystal plane that is tangential to the apex of the interfacial curvature. Consequently, $h_{j^{*}}$ evaluated from this reference plane is taken as the effective crystal truncation.

**Figure 11:**
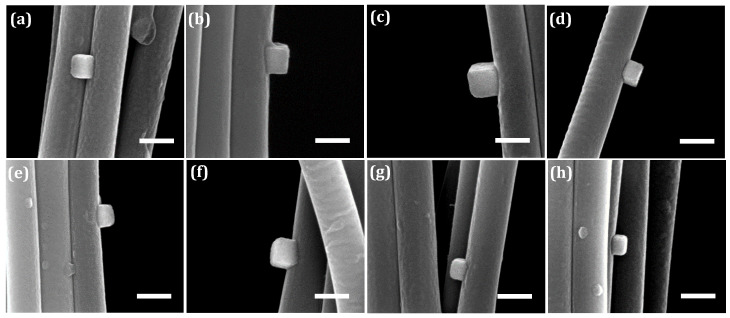
(**a**–**h**) Cubic copper nanostructures exhibiting different levels of substrate-influenced truncation on the nanofibers. Scale bar is 300 nm.

For the computation of the adhesion energy, the approximate elevation profiles of copper nanocubes exhibiting distinct levels of truncation on the nanofibers are shown in Figure 11. These microstructures help to achieve relative accuracy in dimension measurements of the cubic nanostructures.

Previous micrographs have provided strong evidence to infer that the equilibrium shape of the copper nanostructures, if isolated or unattached, is a cubic structure. However, in a stricter sense, accurate structural derivation of the equilibrium shape of the free particle from the particle shape as modified by the substrate can only be made if it contains a Wulff point that also represents a center of inversion symmetry [49]. Otherwise, the crystal shape in general is undefined. As a result, in the characterization of the copper nanostructure on a PAN fiber substrate, we have designated a dimension ratio, i.e., B/A (see Supporting Information), of at least 0.7 to be indisputably indicative of a cubic structure under the reasonable assumption that it contains a bisecting plane of the unattached nanocube, and the Wulff point is at the center of this plane. In addition, the dimension of the non-orthogonal top equilibrium facet becomes the effective cube dimension, since it remains unchanged as the substrate effect is limited to the through-thickness of the crystal [46]. Based on these assumptions, and using the idealized schematic of the cube growth on the PAN fibers as shown in Figure 10c, the distance $h_{j^{*}}$ is expressed as a function of cube dimensions as well as fiber radius (see Figure S4 in supplementary information for derivation of geometric relationships). The surface free energy of {100} copper facets is taken as 1.783 J/m^2^ [50]. Nanocubes on microfibers have been excluded from the truncation analysis due to the ridge-like surface roughness that obstructs clear assessment and evaluation of the interfacial area.

Table 1 shows the summary of analyses of copper nanocube shapes on nanofibers and corresponding adhesion energies as predicted by the Gibbs–Wulff–Kaischew shape theory. The average adhesion energy is 1.08 ± 0.30 J/m^2^.

## 4. Adhesion Energy Qualification and Contextualization

With respect to the energy release rate model for estimation of the adhesion energy, the peculiar substrate geometry of the fibers coupled with the stochastic nature of crystal nucleation and growth precluded the acquisition of more approximate elevation views of cubes exhibiting debonding events, as shown in Figure 8. As a result, the value obtained could not be vetted by rigorous statistical analysis. In addition, while we have utilized the global strain for the adhesion energy computation, neck formation and propagation in the nanofibers can considerably increase the local plastic strain rate [51], and as a corollary, neck strains can be markedly greater than applied strains. Under the assumption of negligible volume changes during deformation and local strain approximation in the necked region based on the reduction in cross sectional area, we obtain adhesion energy of ∼84 J/m^2^. This unusually high value will erroneously subsume the plastic work, which is not accounted for by the model. Hence, the value obtained from the energy release model represent a first order lower bound approximation of the adhesion energy.

A consideration in quantifying the adhesion of the copper nanocubes to both types of fibers is the variability in the stiffness of the fibers that may occur due to processing. While the experimental methodology did not allow direct determination of the mechanical properties of the nanofibers, it is known from the literature that the elastic modulus of PAN nanofibers with diameters similar to those in this study may exceed those of bulk PAN by 10–20% of value stated for computation; this is a direct result of the polymer structure due to processing conditions [52]. Assuming an 18% increase in the modulus of the nanofibers over the bulk value in the current study, the estimated change in adhesion energy (Equation (1)) would be about 15% (an increased stiffness would cause a higher adhesion energy value), bringing our lower bound estimate closer to that determined from the Gibbs–Wulff–Kaischew model. While the current study did not quantify the adhesion on the microfibers using the strain energy release method due to the localized craze formation, it is also possible that those fibers have modulus values that differ from bulk. The centrifugal force generated from drum rotation may promote chain alignment during electrospinning, enhancing mechanical properties [53]. However, temperature gradients induced by the rapid solvent (DMF + acetone) evaporation process, coupled with the microfiber’s smaller surface area for diffusion, may lead to a phase separation, which ultimately creates “locked-in” pores that substantially degrade the mechanical properties of the microfibers [52,54]. Finally, as modulus variations due to polymer structures created with different processing may impact the adhesion, it would also be possible that surface structure would slightly alter the surface energies used in the Gibbs–Wulff–Kaischew model. These coupled uncertainties are one reason for using two complementary methods of quantifying the adhesion energy in this system, and the relative similarity of the adhesion energy between a growth-based and mechanical strain-based method suggests that the values presented here are reasonable first order measurements of the adhesion in this system, and reflect the performance noted in images such as Figure 8, where the metallic cubes clearly are still adhered to highly strained PAN fibers.

For the Gibbs-Wulff-Kaischew shape theory, inaccuracies in the measurement of copper nanocube dimensions represent the major source of uncertainties in the adhesion energy quantification. Nevertheless, with the aforementioned factors as qualifications, the adhesion energy as predicted by the strain energy release model is in good agreement with values predicted for styrene-*co*-acrylonitrile systems and (001) copper facet as obtained with molecular dynamics (MD) simulations (0.51 ± 0.02 J/m^2^) [55]. In addition, the average adhesion energy value as established using the Gibbs–Wulff–Kaischew shape theory is consistent with values obtained for gold delamination on polyimide substrate (∼1 J/m^2^) using a four-point bend testing [56] and for copper films with adhesion-promoting titanium interlayer and stressed chromium overlayers on polyimide substrates (∼1 J/m^2^) using in-situ tensile tests inside a scanning electron microscope [57]. Altogether, these models give useful approximation of the adhesion energy of copper nanostructures on PAN nanofibers.

## 5. Conclusions

Discrete nanostructures on electrospun fibers are essentially consolidated structures of two distinct material classes. Ascertaining the integrity of interfacial adhesion is pivotal for a sustained functional performance. For the purpose of studying interfacial adhesion, we have synthesized well-defined copper nanocubes on PAN nanofibers and microfibers via a solution-phase, bottom-up synthesis method. The nanocube morphology represents a model nanostructure for the study of interfacial adhesion of metallic nanostructures on electrospun fibers, as it affords a definite contact area for microstructural assessment. Micromechanical deformation of these composite structures has revealed the robust adhesion of copper nanocubes on the nanofibers and microfibers. Raman shifts provide strong evidence for chemisorption as the primary anchoring mechanism, and this is believed to be optimized by the fact that crystal growth was based on a solution-based deposition protocol. Finally, the adhesion energy computation using the Gibbs–Wulff–Kaischew shape theory and energy release rate model give a useful first order approximation value of about 1J/m^2^ and a lower bound of 0.48 J/m^2^, respectively, for copper nanocubes on PAN nanofibers with diameters on the order of 300 nm.

## Figures and Tables

**Table 1:** Dimensions of truncated cubes and corresponding adhesion energies based on Equation (S1).

Figure Reference	A (nm)	B (nm)	Adhesion Energy (J/m^2^)	Average Adhesion Energy (J/m^2^)
Figure 3b	223 ± 5	208 ± 3	0.93 ± 0.05	1.08 ± 0.30
Figure 5a	157 ± 2	148 ± 2	0.66 ± 0.004	
Figure 11a	185 ± 4	133 ± 5	1.55 ± 0.03	
Figure 11b	216 ± 4	187 ± 8	1.14 ± 0.04	
Figure 11c	239 ± 3	229 ± 5	0.91 ± 0.02	
Figure 11d	149 ± 3	139 ± 2	0.66 ± 0.03	
Figure 11e	185 ± 5	144 ± 6	1.33 ± 0.02	
Figure 11f	190 ± 6	151 ± 16	1.30 ± 0.20	
Figure 11g	153 ± 5	128 ± 4	1.02 ± 0.02	
Figure 11h	137 ± 5	103 ± 2	1.27 ± 0.06	

## Supplementary Materials

The following are available online at https://www.mdpi.com/2079-4991/10/10/1982/s1, Figure S1: Histogram for distribution of edge lengths on (a) nanofibers and (b) microfibers, Figure S2: Elevation views of copper nanocubes on (a) nanofibers and (b) microfibers, Figure S3: Representative deformation profile of PAN microfibers at strain of 11% (a) incipient craze formation showing transverse crack propagation (b) Highly strained craze fibrils across ruptured microfiber cross sections, Figure S4: Idealized equilibrium shape of a nanocube on a curved fiber surface (a) truncated cube showing distance of bounding facets to Wulff point (b) truncated cube with highlighted Geometric relationships, Table S1: student *t*-test average for edge lengths for nanocubes on the nanofibers and microfibers at α = 0.05.
