# Supplementary material for: Well-Adhered Copper Nanocubes on Electrospun Polymeric Fibers
Source: Nanomaterials (Basel). 2020 Oct 7;10(10):1982. doi: 10.3390/nano10101982 (PMC7601943; doi:10.3390/nano10101982)
Supplement: Supplementary file 1 [file nanomaterials-10-01982-s001.pdf]

SUPPORTING INFORMATION

Well-Adhered Copper Nanocubes on Electrospun Polymeric Fibers

Temitope Q. Aminu [1], Molly C. Brockway [2], Jack L. Skinner [2] and David F. Bahr [1]

[1] School of Materials Engineering, Purdue University, West Lafayette, IN 47907, USA; taminu@purdue.edu
[2] Mechanical Engineering, Montana Technological University, Butte, MT 59701 USA; mbrockway@mtech.edu(M.C.B.); jskinner@mtech.edu(J.L.S.)
* Correspondence: dfbahr@purdue.edu

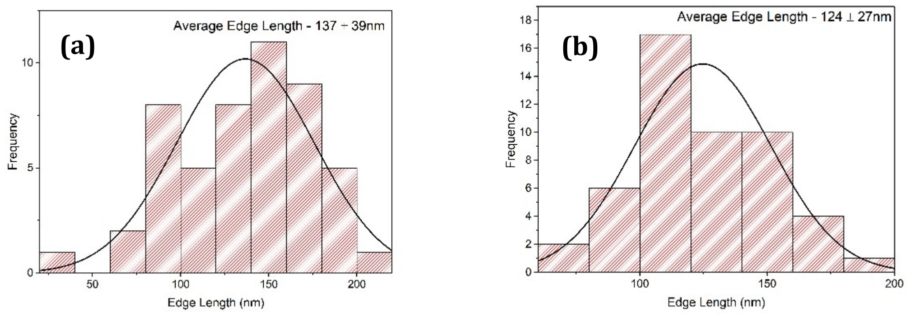

Figure S1: Histogram for distribution of edge lengths on (a) nanofibers (b) microfibers

Table S1: Student T-test average for edge lengths for nanocubes on the nanofibers and microfibers for α =0.05

t Statistic	Prob > |t|
1.79789	0.07568

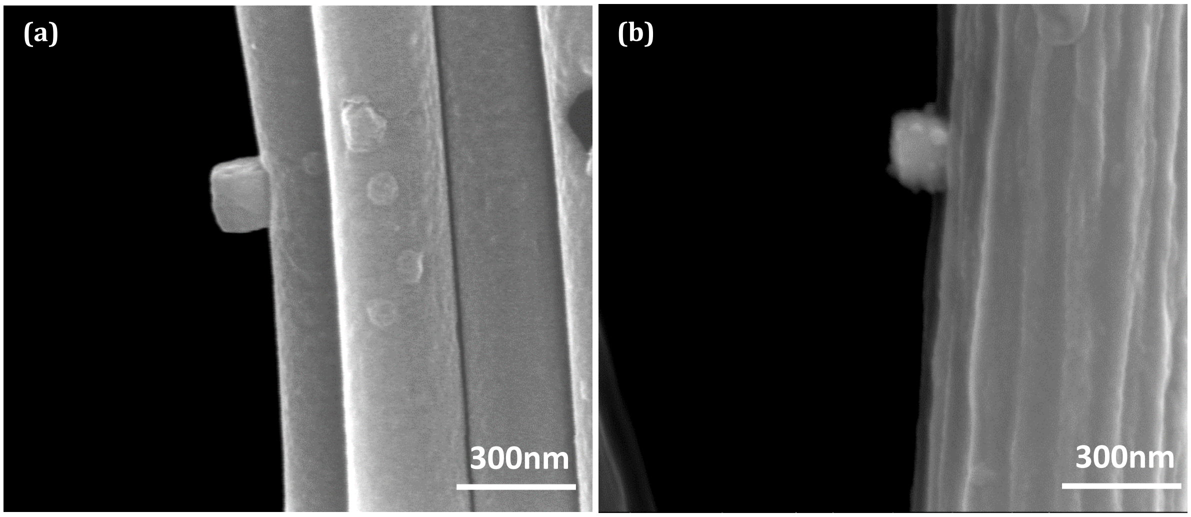

Figure S2: Elevation views of copper nanocubes on PAN (a) nanofibers (b) microfibers

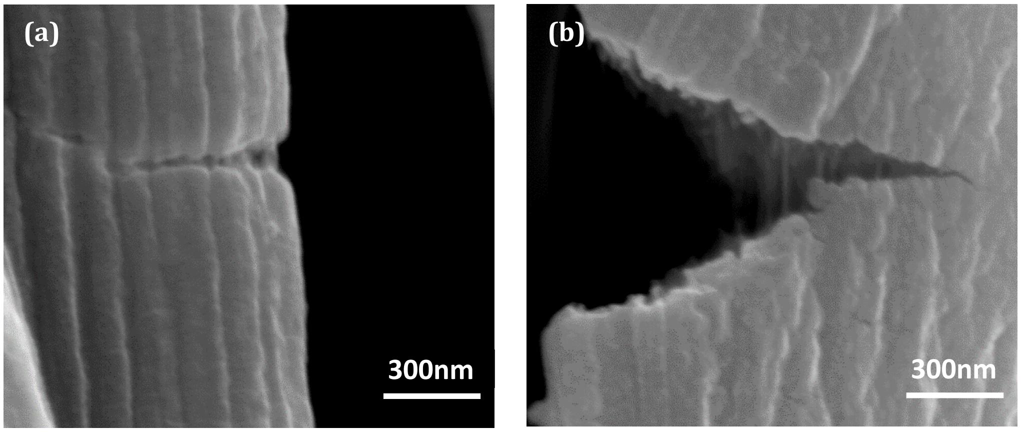

Figure S3: Representative deformation profile of PAN microfibers at strain of 11% (a) incipient craze formation showing transverse crack propagation (b) Highly strained craze fibrils across ruptured microfiber cross sections

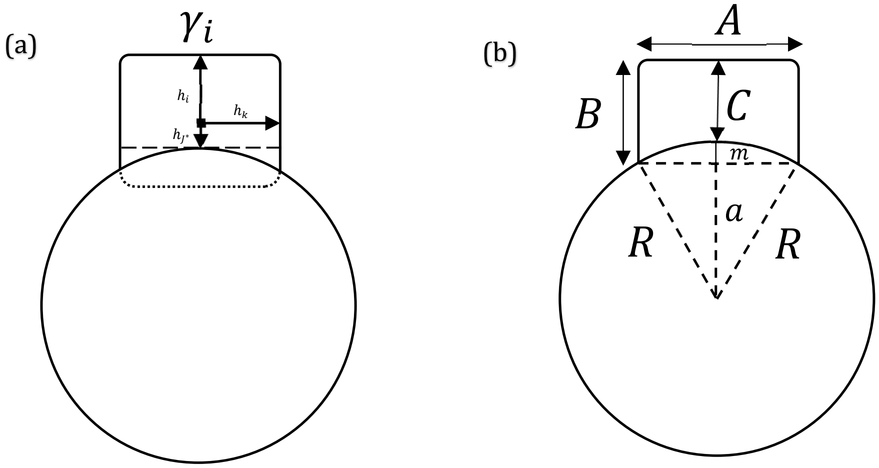

Figure S4: Idealized equilibrium shape of a nanocube on a curved fiber surface (a) truncated cube showing distance of bounding facets to Wulff point (b) truncated cube with highlighted Geometric relationships

$$C = h_i + h_{J^*}$$

Based on the assumption of the Wulff point present in the equilibrium shape on the substrate, and being a center of inversion symmetry

$$h_i = \frac{A}{2} \qquad\qquad h_{J^*} = C - \frac{A}{2}$$

From the right triangle formed by half–length of the cube, fiber radius and geometric construct 'a', 'a' can be expressed as

$$a = \sqrt{R^2 - \left(\frac{A}{2}\right)^2}$$

Since,

$$R = a + m$$

$$m = R - \sqrt{R^2 - \left(\frac{A}{2}\right)^2}$$

and

$$C = B - m$$

then,

$$h_{J^*} = \mathrm{B} - \frac{A}{2} - R + \sqrt{R^2 - \left(\frac{A}{2}\right)^2}$$

Simplifying further

$$h_{J^*} = \frac{2(B - R) - A + \sqrt{(4R^2 - A^2)}}{2}$$

From the Gibbs-Wulff-Kaischew shape theory

$$\frac{\gamma_i}{h_i} = \frac{\gamma_j - \beta}{h_{j^*}}$$

And taking the assumption that,

$$\gamma_i = \gamma_j$$

Adhesion Energy of the nanocubes on the fibers is given as

$$\beta = \gamma_i - \gamma_i \left(\frac{2(B - R) - A + \sqrt{(4R^2 - A^2)}}{A} \right) \tag{S1}$$
